# Plexiform neurofibroma in the submandibular gland along with small diffuse neurofibroma in the floor of the mouth but without neurofibromatosis-1: a rare case report

**DOI:** 10.3332/ecancer.2013.313

**Published:** 2013-05-02

**Authors:** Hemlata T Kamra, Sunita S Dantkale, Khushboo Birla, Pankaj W Sakinlawar, Paresh H Bharia

**Affiliations:** Department of Pathology, Government Medical College, Latur-413512, Maharashtra, India

**Keywords:** Plexiform, submandibular gland, neurofibroma, mouth

## Abstract

Plexiform neurofibroma is more commonly seen in the orbit, neck, back, and inguinal region. It is extremely rare in the submandibular gland. These lesions rarely transform into malignancy but are locally infiltrative and can lead to haemorrhage. Therefore, plexiform neurofibroma should always be considered during differential diagnosis while excising a submandibular gland mass. We present here a case of plexiform neurofibroma in the submandibular gland and diffuse neurofibroma in the floor of the mouth in a 27-year-old female, not associated with neurofibromatosis-1.

## Introduction

Neurofibroma is a disease of the peripheral nervous system and occurs most commonly in the extremities. Several forms have been described: cutaneous neurofibromas (both localised and diffuse types), intraneural neurofibromas (localised and plexiform), massive soft tissue neurofibromas (solitary or multiple), and sporadic neurofibromas or those associated with neurofibromatosis-1 (NF-1) [1, 2]. Plexiform neurofibroma is usually recognised as a pathognomonic criterion of NF-1 (or Von Recklinghausen’s disease); it may also occur as a solitary lesion arising in a nerve root [3]. Plexiform neurofibromas of the submandibular gland are extremely rare. To date, only seven cases have been reported in the literature, and only one case was not associated with NF-1 [4, 5].

## Case Report

**Figure 1:**
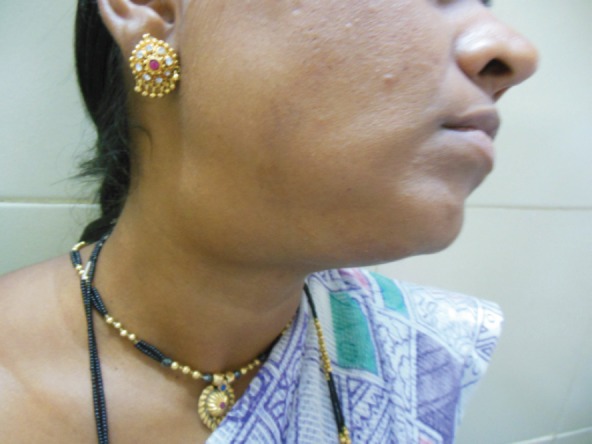
Photograph of patient with submandibular swelling.

**Figure 2:**
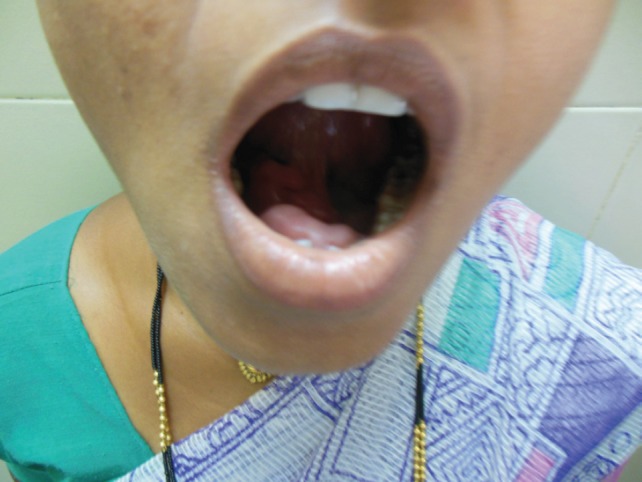
Photograph of patient with tongue lifted showing swelling in the floor of the mouth.

**Figure 3:**
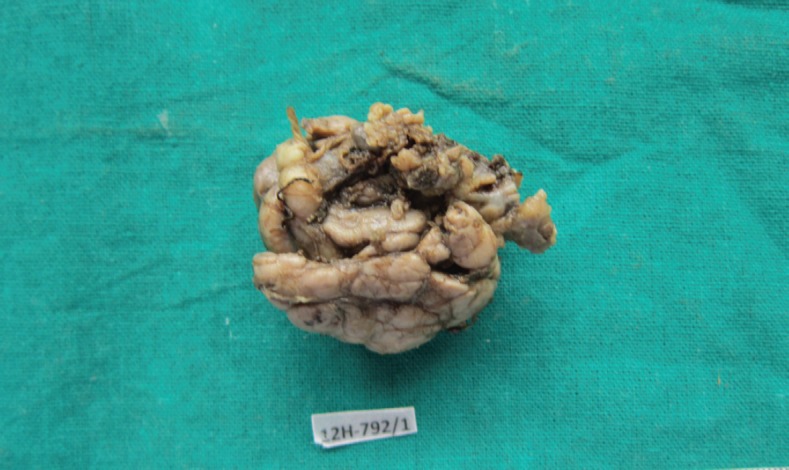
Photograph of the submandibular mass—grey white, irregular, capsulated, and nodular (4608 × 2742 pixels).

**Figure 4:**
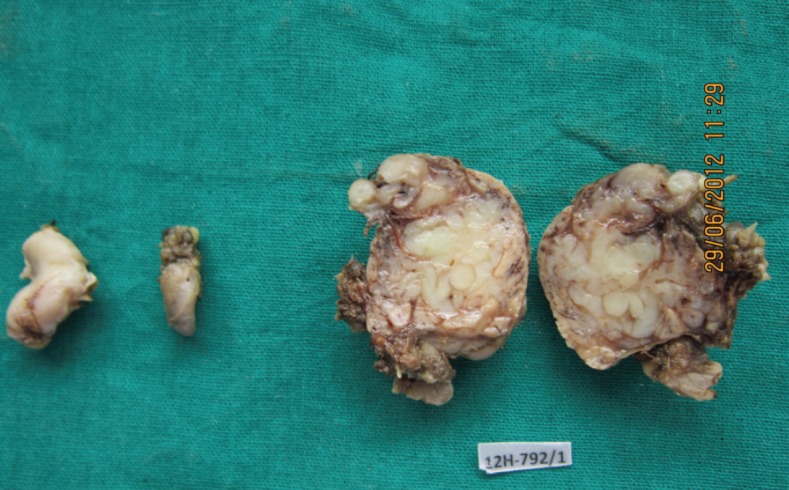
Photograph showing the swelling from the floor of the mouth, the lymph node, and the cut section of the submandibular mass (4608 × 2765 pixels).

**Figure 6:**
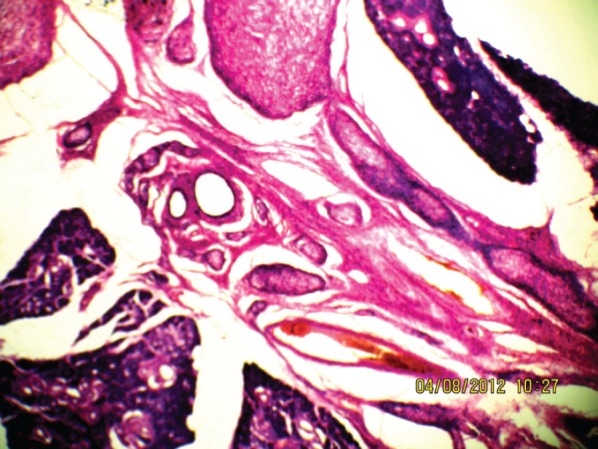
The plexiform neurofibroma between the submandibular gland (10Х, 3264 × 2448 pixels, H&E stain).

**Figure 7:**
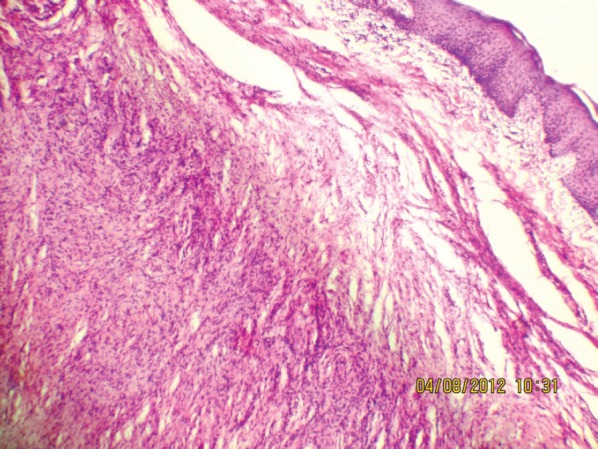
Interlacing bundles of elongated cells with wavy nuclei in dermis (10Х, 3264 × 2448 pixels, H&E stain).

**Figure 8:**
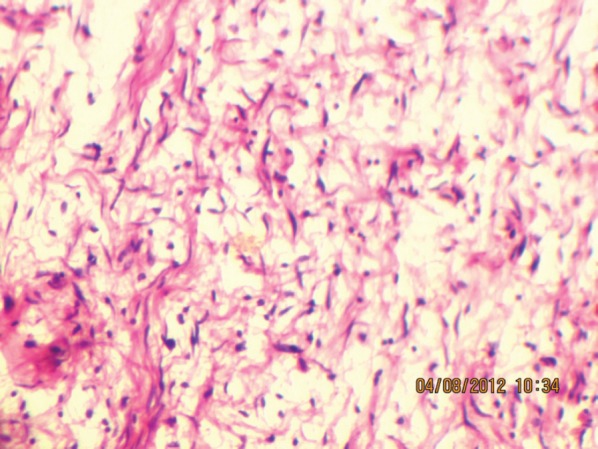
Wavy nuclei in a myxoid background (40Х, 3264 × 2448 pixels, H&E stain).

A 27-year-old female presented in the surgical outpatient department with swelling in the left submandibular region and the floor of the mouth that had been present for six months. The swelling had increased gradually in size. The patient complained of difficulty in eating food. On physical examination, there was a non-tender mass, firm in consistency, not attached to the skin, measuring 5 × 4 cm in the submandibular region (Figure 1) and 2.5 × 1 cm in the floor of the mouth (Figure 2). No nerve involvement was observed. On systemic examination, there was no swelling or café-au-lait spots all over the body. A family history of NF was absent. Laboratory investigations were within normal limits. An ultrasound of both masses revealed heterogenous echogenecity with ill-defined borders. The fine-needle aspiration cytology (FNAC) of the submandibular mass was inconclusive, but the FNAC of the swelling in the floor of the mouth revealed bland spindle cells in a haemorrhagic aspirate. A CT scan of the swelling in the submandibular region and the floor of the mouth revealed hypoattenuated masses. Excision of both the swellings was done simultaneously under general anaesthesia. The origin of the tumour was not obvious at surgery. The submandibular gland mass was an irregular, nodular, capsulated grey-white piece of tissue measuring 4.5 × 3 × 2 cm; it was firm in consistency (Figure 3). The cut section was lobulated, with a white, hyalinised appearance. The mass from the floor of the mouth was an irregular white piece of tissue measuring 2 × 0.6 × 0.3 cm; it was firm in consistency and a small lymph node measuring 1 × 0.5 cm. The cut section was homogenous, grey-white, and glistening (Figure 4). On examination under low-power magnification (10X), the submandibular gland mass was nodular and showed a tortuous mass of expanded nerve branches, which are seen cut in various planes of section. (Figure 5a and 5b). The tumour was comprised of wavy serpentine nuclei with pink cytoplasm and a myxoid background, with areas of collagen bundles, and it was infiltrated by mild lymphocytic infiltrate. A diagnosis of plexiform neurofibroma was obvious on microscopic examination (Figure 6). Histopathological examination of the mass in the floor of the mouth showed tissue lined by nonkeratinised stratified squamous epithelium. The subepithelial tissue revealed a tumour comprised of interlacing bundles of elongated cells with wavy nuclei, intimately associated with wire-like strands of collagen (Figure 7). The background showed a myxoid appearance (Figure 8). A diagnosis of diffuse neurofibroma was made. Although, immunohistochemically, the tumour was positive for S-100 protein, the diagnosis was obvious on light microscopy.

## Discussion

Neurofibromas constitute 0.4% of all salivary neoplasms. Plexiform neurofibroma is mostly seen in the parotid gland and is very rare in the submandibular salivary gland [5]. Neurofibromas demonstrate diffuse cylindrical enlargement of multiple fascicles of a nerve, including the nerve branches leading to a diffuse mass of thickened nerves [6], and the gross pathological appearance is referred to as a bag of worms. The common sites involved are the major nerve trunks of the head and neck region because of the rich innervations of this area [7]. Wilkinson *et al *have reported plexiform neurofibroma in the bladder [8]. Neurofibromas are slow growing, grow along nerves, and are locally infiltrative. Plexiform neurofibroma is frequently seen in patients with Type 1 NF, but solitary plexiform neurofibroma has also been occasionally reported.

Histologically, neurofibromas consist of Schwann cells, nerve fibres, mast cells and perineural and endoneurial fibroblasts in a myxoid matrix. At CT scan, neurofibromas are round-to-oval well-defined homogenous masses that have an attenuation value of 22–25 HU on non-enhanced scans and 30–50 HU after contrast material administration. The low attenuation is attributed to high lipid or water content within the mucinous matrix, entrapment of perineural adipose tissue, and less often, cystic degeneration [9]. On T1-weighted MR images, they are isointense to hypointense when compared with muscle signal intensity. These masses may show a target sign on T2-weighted images with peripheral hyperintense signal intensity and central isointense to hypointense signal intensity. Contrast enhancement may be heterogenous [9]. On the other hand, superficial neurofibromas are asymmetric, have non-targetlike signal intensity, lack nodular or fascicular morphology, and are likely to involve skin [10].

Microscopically, there are widespread spindle cells with ovoid to thin and elongated bland nuclei with minimal or no pleomorphism and without prominent nucleoli. The proportion of collagenous to myxomatous component is variable.

Plexiform neurofibromas represent 14% of all benign mesenchymal tumours and 10% of non-epithelial salivary gland tumours [11]. Although they are benign, they have a 2%–5% potential for malignant transformation [12]. Patients with NF-1 and plexiform neurofibromas have a higher mortality rate when compared with patients with or without asymptomatic plexiform neurofibromas [13]. Research on the biologic basis of plexiform neurofibroma in NF-1 states that increasing evidence of loss of NF-1 expression in neoplastic Schwann cells is associated with elevated levels of activated ras, supporting the notion that the NF-1 gene product neurofibromin acts as a growth regulator by inhibiting ras protein activity (correlates with human cancer development and dysfunction of neurofibromin) [14]. Solitary plexiform neurofibroma could be a clinical manifestation of segmental NF resulting from mosaicism of NF-1 [15]. Although genetic testing for some of the mutations of the NF-1 gene is available, there is no evidence that such testing is helpful in diagnosing NF-1 in patients with isolated plexiform neurofibroma [14]. Immunohistochemically, the tumour is positive for anti-S 100 protein.

In our case, the patient had no features of the seven criteria used to diagnose NF but had two neurofibromas with different morphologies. The two lesions may be the result of hyperplasia of different small nerve trunks. The patient was advised to receive but did not undergo genetic evaluation. Surgical excision is probably the only therapy. Recurrence is reported in 20% of the patients with plexiform neurofibroma after complete resection and increases to 44% with incomplete resection (when there is involvement of vital structures) [9].

## Conclusion

The diagnostic yield of fine-needle aspiration of plexiform neurofibroma of the submandibular gland is low, hence diagnosis is based on histopathological examination. In a solitary neurofibroma occurring without stigmata of NF-1, the tumour probably represents the segmental form of NF-1 caused by a later somatic mutation. In adults, plexiform neurofibroma should be considered in differential diagnosis of isolated swellings, although it is of neurogenic rather than salivary gland origin. Follow-up of the patients is necessary due to the possibility of recurrence and malignant transformation. Genetic counselling and evaluation of other family members should be performed for those suspected to be affected by the syndrome.

## Figures and Tables

**Figure 5a:**
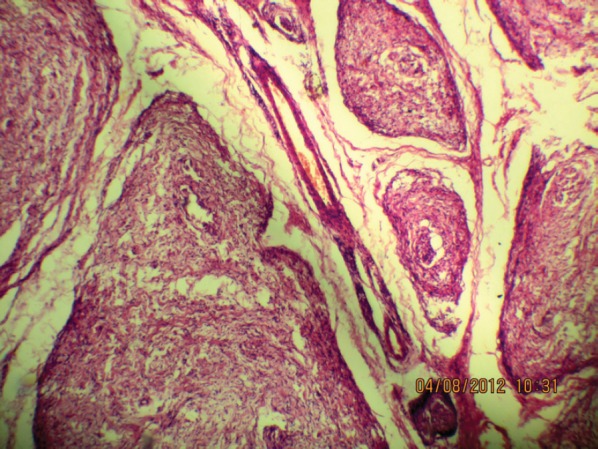
The tortuous mass of expanded nerve branches are seen cut in various planes (10Х, 3264 × 2448 pixels, H&E stain).

**Figure 5b:**
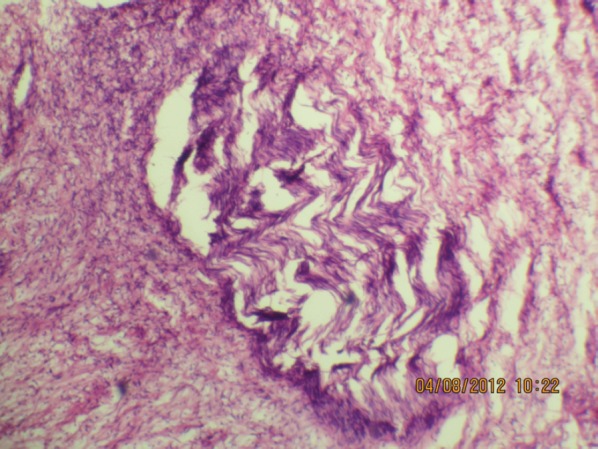
The expanded nerve branch (40Х, 3264 × 2448 pixels, H&E stain).
